# Combined autologous and allograft limbal cell transplantation with penetrating keratoplasty in a case of chemical corneal burn patient

**DOI:** 10.4103/0974-620X.57312

**Published:** 2009

**Authors:** Sandip Mitra

**Affiliations:** Department of Ophthalmology, Al-Zahra Private Hospital, Sharjah, UAE

**Keywords:** *Limbal stem cell transplant*, *Allograft*, *autologous*, *chemical burns*, *limbal stem cell transplant*

## Abstract

A patient with chemical corneal burn presented two months after the acute episode of chemical injury to his right eye (OD) and was diagnosed with severe limbal stem cell deficiency and with vascularized corneal opacity OD. Limbal cell transplantation and penetrating keratoplasty (PKP) was performed. The autologous and allograft limbal tissue included peripheral cornea, limbus and conjunctiva obtained from contralateral eye and cadaveric eye bank cornea. Central corneal button was used for a PKP with 32 intermittent sutures. One year after the procedure, the corneal surface remains clear with a best corrected visual acuity of 6/12 (-2.00 DS / -2.75 DC-/ 150°. Eighteen sutures are still in place; no vascularization has extended beyond the host graft junction. Ocular surface is wetting well with no filamentary keratitis.

Combined autologous and allograft limbal cell transplant can be performed for severe deficiency of corneal stem cells in a patient with chemical corneal burn.

## Introduction

Chemical burns of the eye are one of the leading causes of visual disability. Chemical burns, particularly alkali burns lead to gross limbal stem cell deficiency.[1] Limbal deficiency, or loss of stem cells, is associated with conjunctivalization of the corneal surface, recurrent and persistent epithelial defects, chronic inflammation, scarring and ulceration of cornea. Keratoepithelioplasty was proposed by Thoft,[2] as a procedure to reconstruct the corneal surface in patients with corneal stem cell deficiency. Subsequently the same author modified the technique to include the limbal tissue, acknowledging the importance of stem cell transplantation in chemical burn. Limbal transplant as proposed by Kenyon and Tsang[3] is probably the best current option for ocular surface reconstruction in the patients with corneal stem cell deficiency.

This case report describes a patient with alkali burn induced severe stem cell deficiency and vascularized corneal opacity, treated with combined autologous and allograft limbal cell transplantation and penetrating keratoplasty.

## Case Report

A 24-year-old patient sustained alkali burn two month prior to his presentation to our clinic. He had been managed with topical antibiotics, lubricants and steroid. Amnion grafting had been done to protect and heal the corneal surface. At presentation, he complained of severe pain, redness, watering and poor vision in the right eye (OD). There was no significant past history of ocular or systemic disease.

**Figure 1:**
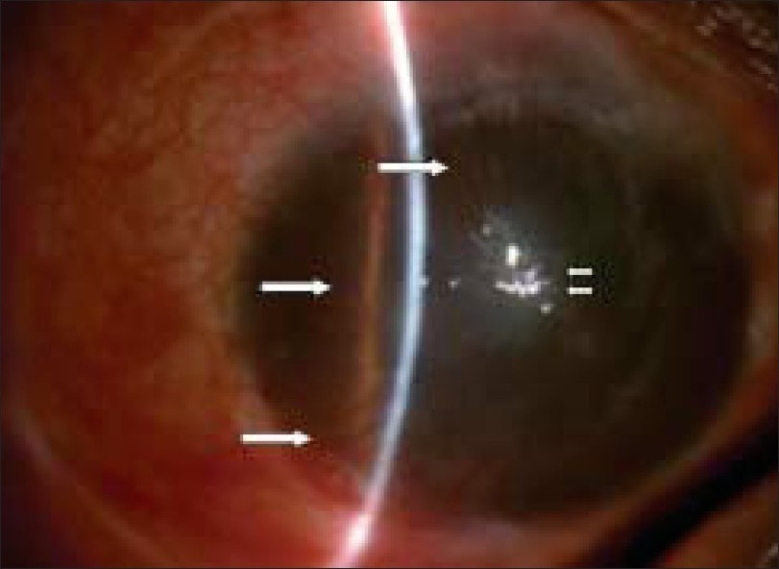
Preoperative photograph of the eye of the patient with alkali burn showing superficial and deep corneal vascularization (single white arrow) with central corneal edema and opacity (double white arrows).

**Figure 2:**
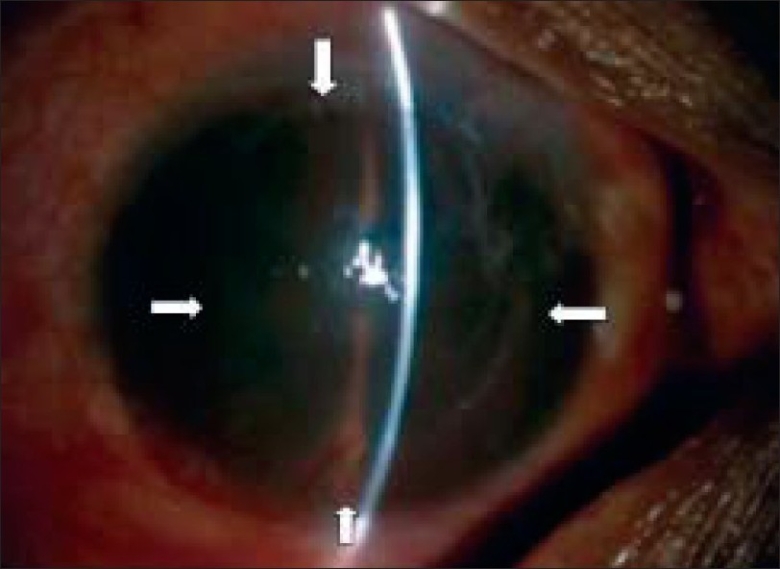
Preoperative picture shows 360 degree of corneal vascularization and stem cell deficiency with limbal ischemia (single white arrow).

Ophthalmic examination revealed an unaided vision of 6/60 OD with no further improvement with refraction. There was narrowing of the palpebral aperture with fornix cicatrization. Slit lamp examination revealed bulbar and ciliary congestion, 360° superficial and deep corneal vascularization with vessels extending 4–5 mm from the limbus into the center of the cornea [Figures 1 and 2]. The central cornea was thickened with epithelial and stromal edema. Fluorescein staining revealed central epithelial defect of 2 mm. Pupil reacted well to both direct and consensual light stimulation and lens appeared clear. There was patchy iris atrophy and intraocular pressure (IOP) was recorded at 20 mm Hg with a Tonopen. Dilated fundus examination revealed normal optic disc, macula and retinal background. Schirmer′s test type I and II was abnormal and tear break-up time (BUT) was reduced.

Pre-operatively, anesthetic clearance was obtained and informed consent was taken from the patient and all surgical risks were explained to the patient including graft rejection.

The surgical technique included the following steps:

- The donor tissue consisting of corneal-limbal-conjunctival explant (2 mm of peripheral cornea, limbus, and 3 mm of bulbar conjunctiva), 2–clock hours each (11–1 o′clock and 5–7 o′clock hours), were harvested from the contralateral eye and the eye bank cornea.
- An 8.5 mm donor corneal graft was obtained from the same donor eye bank cornea.
- Limbal stem cell harvest: A double-edged calibrated diamond knife was set to 125 µm, and used to make a circumferential corneal incision parallel to the limbus, and two radial incisions were then made extending from either ends of the limbal tissue to beyond the limbus under the conjunctiva. Three millimeters of the conjunctiva, attached to the corneal and limbal explants along the limbal border, was then excised using a Barron Hess burg disposable donor punch to obtain an 8.5 mm donor corneal button. The peripheral corneal and conjunctiva tissue from the eye bank cornea was used to develop a 3 × 5 mm corneal–limbal–conjunctival graft tissue using the degraded diamond knife and lamellar dissector.
- Preparation of recipient bed: The conjunctiva at the limbus was incised 360° and hemostasis was achieved using bipolar cautery. Abnormal corneal epithelium and the superficial fibro-vascular scar tissue were removed using a blunt dissector. The diamond knife was set at 75 microns and a bed corresponding in dimension to the donor explants was fashioned at the recipient site(s). The donor limbal graft was sutured onto the recipient eye with two interrupted 10-0 monofilament nylon sutures at the corneal margin and two along the scleral edge of the explants. The conjunctiva of the recipient eye was approximated to the donor conjunctiva with 8-0 Vicryl sutures, taking a bite into the episclera. An 8 mm Barron Hessburg punch was used to make a corneal punch at the recipient site and anterior chamber was entered using a #15-blade. The corneal button was removed using corneal scissors. The 8.5 mm donor cornea was then sutured to the recipient bed using 32 interrupted 10-0 monofilament nylon sutures.

Postoperatively, topical prednisolone 1% eye drops four times; moxifloxacillin eye drops four times, preservative free topical lubricants and ointment TobraDex at night was commenced.

Oral prednisolone (1 mg/kg body weight) was administered for 1 week and tapered over 6 weeks. Follow-up checks were performed on day 1, 2, 3, 4, 10 and 14, and then after 1 month, and monthly for 1 year. Sutures were removed 4 months postoperatively, whenever they got loose and collected mucus on them, unaided and best-corrected vision, slit lamp examination, IOP measurement using the Tonopen and dilated fundus examination was performed during each visit.

**Figure 3:**
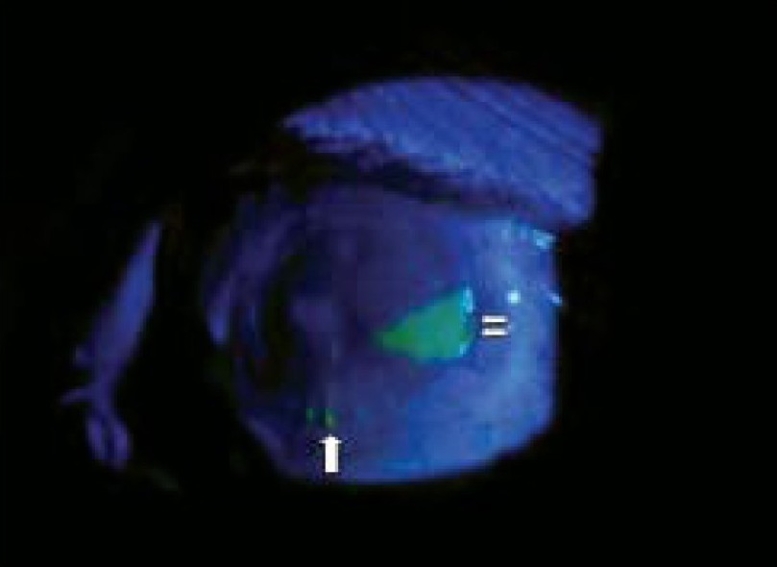
At two weeks follow-up, Cobalt blue light examination reveals almost 90% epithelial healing with small epithelial defect at three o′ clock, staining with fluorescein (double white arrows) and two loose sutures at six o′ clock (single white arrow).

**Figure 4:**
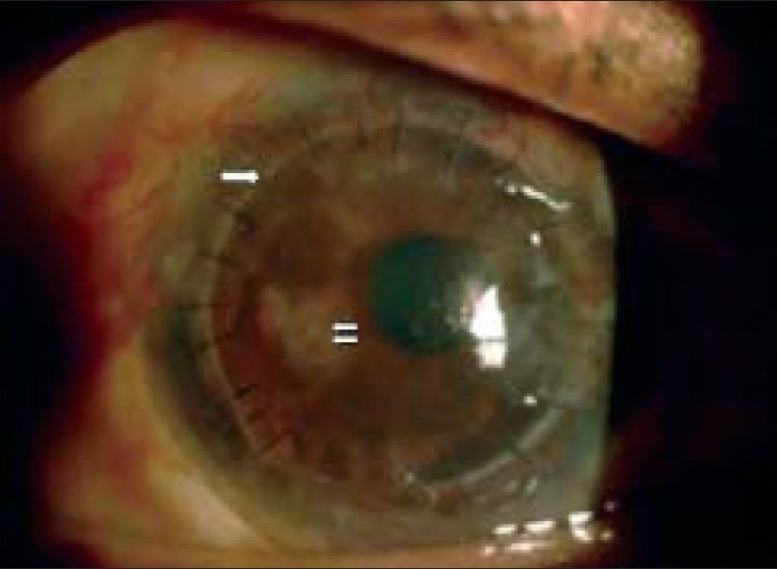
At 12 months follow-up shows clear corneal graft with good limbal stem cells and vascularization not crossing the graft host junction (single arrow). There were areas of patchy iris atrophy suggesting anterior segment ischemia due to chemical burn (double white arrows).

No intraoperative complications occurred. Postoperatively, the limbal graft showed epithelial outgrowths within the first two days and almost 90% corneal surface was completely epithelialized within two weeks [Figure 3]. There was no infection, limbal graft failure or slippage of tissue. The epithelium was stable, without recurrences of epithelial defects, or corneal neovascularization. The vision improved to 6/36 unaided and 6/12 with glasses (-2.00 DS/ -2.75 DC × 150°) by six months. At the final postoperative visit, 18 sutures were in place. No vascularization was observed beyond the host graft junction [Figure 4]. The mean IOP remained normal at 16 mm Hg. No cataract has been observed. Ocular surface was wetting well with no filamentary keratitis.

## Discussion

Various techniques of limbal transplantation have been reported. All these procedures remove the host′s altered corneal epithelium and provide a new source of epithelium for the diseased ocular surface. From the donor tissue obtained from the fellow eye (auto graft)[4] and/or cadaveric whole globe or corneoscleral rim of the eye bank cornea (allograft), transient amplifying cells are generated which migrate onto the denuded corneal surface of the host.[5] Limbal transplant procedures also vary depending on the carrier tissue used for the limbal stem cells. Carrier tissue is needed in limbal transplantation because it is not possible to transfer limbal stem cells alone. Either conjunctiva (conjunctival limbal graft) or corneal/limbal stroma (keratolimbal graft) have been used as carrier tissue for limbal stem cells.

This study describes a patient who sustained alkali burn OD, developed severe limbal ischemia and corneal vascularization with repeated corneal epithelial defect and underwent a combination of auto and allograft limbal stem cell transplant along with penetrating keratoplasty (PKP). In this case, the clinical features were suggestive of limbal stem cell ischemia; however, an impression cytology for Periodic Acid Schiff stain (PAS) for conjunctival goblet cells and immunohistochemistry analysis for cytokines CK 19 and CK 3 confirms limbal cell ischemia.[6‐7] Superficial corneal stroma, perilimbal sclera and conjunctiva were used as carriers of limbal stem cells and providers of an adequate microenvironment for their survival and replication. Preservative free topical drops were administered along with lubricants to aid the migration of the limbal stem cell over the corneal graft. Use of soft contact lens was avoided due to the risk of infection and poor retention of the lens due to irregular corneal surface. Survival of the limbal stem cells in allograft transplantation is improved by selecting live related donor with HLA class I and II matched.[8] The patient achieved rapid surface healing with restoration of a smooth, stable and optically improved surface, resulting in improved visual acuity and better graft survival with decreased graft rejection. However, due to nonavailability of the live-matched donor and technical difficulties, the allograft was obtained from a cadaveric eye, which showed excellent survival so far. We used topical and systemic steroid to suppress the immunity and allow the persistence of the donor epithelial cells. However in case of recurrent inflammations one can use systemic cyclosporin, azathioprine, tacrolimus or mycophenolate.[9‐10] However, these drugs are expensive and require constant watch for side effects like hypertension and renal toxicity.

In conclusion, the use of autologous and allograft limbal transplantation with corneal, scleral and conjunctival carriers was useful for ocular surface reconstruction and graft survival in-patient with chemical burn and severe limbal stem cell loss.

Adequate immunosuppression was required in the initial period to prevent graft rejection.
